# Evidence of a reduced role for circumscribed interests in the social attention patterns of children with Autism Spectrum Disorder

**DOI:** 10.1007/s10803-022-05638-4

**Published:** 2022-08-04

**Authors:** Z Ambarchi, K. A. Boulton, R. Thapa, E. E. Thomas, M. M. DeMayo, N. J. Sasson, I. B. Hickie, Adam J. Guastella

**Affiliations:** 1 Brain and Mind Centre, Children’s Hospital Westmead Clinical School, Faculty of Medicine and Health, University of Sydney, 2050 Sydney, Australia; 2 Brain and Mind Centre, Central Clinical School, Faculty of Medicine and Health, University of Sydney, Sydney, Australia; 3 Department of Psychology, School of Behavioral and Brain Sciences, The University of Texas at Dallas, Richardson, USA

**Keywords:** Autism spectrum disorder, Circumscribed interests, Eye-tracking, Social attention, Object attention, Gaze

## Abstract

Reduced social attention is characteristic of Autism Spectrum Disorder (ASD). It has been suggested to result from an early onset and excessive influence of circumscribed interests (CIs) on gaze behaviour, compared to typically developing (TYP) individuals. To date, these findings have been mixed. The current eye-tracking study utilised a visual preference paradigm to investigate the influence of CI versus non-CI objects on attention patterns in children with ASD (aged 3–12 years, n = 37) and their age-matched TYP peers (n = 30). Compared to TYP, social and object attention was reduced in the ASD group irrespective of the presence of CIs. Results suggest a reduced role for CIs and extend recent evidence of atypical attention patterns across social and non-social domains in ASD.

Atypicalities in attention to social information is central to diagnostic criteria for autism spectrum disorder (ASD). The early-age onset and potential cascading effects on social communication and functioning skills has led to a proliferation of research investigating gaze behaviour as a biomarker for early diagnosis and as a potential target for intervention (Bradshaw et al., 2019; Frye et al., 2019; Guastella et al., 2008; Shic, 2016; Webb et al., 2020). Research utilising eye tracking technology has revealed reduced social attention across a range of gaze behaviours, such as the duration of fixations to the eyes and face (Frazier et al., 2017; Klin et al., 2002), the processing of faces and emotional expressions (Black et al., 2017; Dawson et al., 2005), the exploration of, and disengagement from, social stimuli (Chawarska et al., 2010; Sasson et al., 2008), orienting to gaze and gaze following (Senju, 2004; Gillespie-Lynch, 2013), joint attention (Franchini et al., 2017), and social cueing (Chevallier et al., 2013). Reduced attention to social cues has been demonstrated in infants as young as six months of age (Chawarska et al., 2013), across childhood and also throughout adulthood (Chita-Tegmark, 2016; Frazier et al., 2017). Moreover, there has been growing speculation that reduced attention may contribute to the observed difficulties in social and adaptive functioning (Klin et al., 2002; Poon et al., 2012; Rice et al., 2012; Tang et al., 2017).

There has, however, been much debate about the extent to which differences in social attention are moderated by context and their specificity to social cues. For example, circumscribed interests (CIs), considered to be a factor in the characteristic restrictive and repetitive behaviour profile in ASD (South et al., 2005; Turner-Brown et al., 2011), have been reported to induce biases in visual attention patterns across childhood in ASD (Elison et al., 2012; Sasson et al., 2011; Sasson & Touchstone, 2014). Sasson and Touchstone (2014) investigated the influence of objects of circumscribed or High Autism Interest (HAI; for example, transportation vehicles, mechanical instruments) and Low Autism Interest (LAI; for example, household items, plants) on social attention patterns in thirty young children with and without ASD. They developed a visual preference task involving a series of 20 images of a face paired with either a HAI or LAI object and found that compared to typically developing (TYP) children, children with ASD were slower to orient to and maintain attention to faces when they were paired with HAI objects, however there were no group differences in social attention in the presence of LAI objects. The ASD group in this study also exhibited a greater preference to attend to HAI relative to LAI objects, complementing findings from earlier studies demonstrating an early-onset and discrete preference in children with ASD to explore and perseverate their attention on CIs in comparison to their TYP peers (Elison et al., 2012; Sasson et al., 2011). In a follow-up study with 87 school-age children aged 6 to 10 years, reduced social attention in the presence of HAI was also demonstrated, however this finding was specific only for male participants with ASD; the attention patterns of female participants with and without ASD were not significantly different (Harrop et al., 2018b). In addition to potential phenotypic variations across sexes, the results of these studies suggested that CIs may moderate attention patterns to both social and non-social elements of a scene from an early age in life and therefore potentially represent an important characteristic in children with ASD.

Other studies investigating the unique influence of CIs on social attention patterns in children with ASD however, have led to variable findings. The visual preference task described above was implemented in a study by Unruh et al., (2016) with results indicating that both adolescents with ASD (n = 41) and their TYP (n = 34) peers demonstrated a preference to attend to HAI compared to LAI objects and reduced social attention in the presence of HAI objects. Furthermore, analysis of between-group differences revealed a preference to look at both object types (HAI and LAI) in the ASD group, while the TYP group preferred to look at faces. A similar finding was reported by Harrison & Slane (2020), with reduced attention to faces in children and adolescents with ASD (n = 16) across object types, and interestingly, a variable influence of HAI on social attention in the TYP (n = 20) but not the ASD group (n = 16). These results are consistent with other eye-tracking studies reporting a similar influence of CIs in ASD and TYP participants, concurrent with reduced social attention in participants with ASD specifically, throughout development (DiCriscio et al., 2016; Mo et al., 2019; Traynor et al., 2019). Findings of reduced social attention in ASD independent of the influence of CIs across childhood lend support to the social motivation hypothesis, which posits an intrinsic, early-onset impairment in the motivation to attend to and engage with socially relevant stimuli, leading to reduced social learning experiences and cascading effects in overall socio-cognitive and social skill development (Chevallier et al., 2012).

Research exploring the relationship between social attention patterns and overall social functioning have led to similarly equivocal findings. There have been some longitudinal studies reporting a relationship between reduced social attention in infancy and poorer language and theory of mind skills in early childhood (Brooks & Meltzoff, 2015; Poon et al., 2012), however other studies in older children with ASD have not yielded significant associations between social attention and social functioning measures (Fujioka et al., 2020; Unruh et al., 2016; van Rijn et al., 2019). Interestingly, the landmark study by Klin and colleagues (2002), as well as Rice et al., (2012) found that greater attention to objects rather than reduced attention to social stimuli, was associated with greater social impairment, while Sasson (2008) reported a positive correlation between exploration of object stimuli and social impairment. The variability in correlational findings between social attention and functioning suggests further exploration inclusive of non-social attention patterns is warranted. Consistent with the social motivation hypothesis, the imbalance of attention to objects over social stimuli has similarly been theorised to lead to fewer social learning experiences and therefore facilitative of the day-to-day social challenges experienced by individuals with ASD (Sasson et al., 2008).

There are equivocal findings across previous studies, suggesting on one hand that the excessive influence of CIs determines the variability in social attention patterns in ASD, and on the other hand, that social attention in this population is reduced irrespective of stimuli and context. This presents a need to better understand how CIs influence the attention patterns of children with ASD and their TYP peers. Thus, the goal of this study was to investigate the influence of CIs on social and object attention patterns in children with ASD compared to TYP peers using established task and outcome measures, and to explore the relationship between these patterns and social functioning. An established visual preference task was employed to facilitate comparability with past research. Based on recent findings, it was hypothesised that a reduction in social attention would be evident in the ASD group regardless of the presence of CIs. It was also hypothesised that both ASD and TYP groups would exhibit greater attention to HAI (i.e., CIs) compared to LAI objects. No hypotheses were made regarding the relationship between attention and social functioning given the variability in findings across studies (Klin et al., 2002; Poon et al., 2012; Rice et al., 2012; Sasson et al., 2008; Unruh et al., 2016; van Rijn et al., 2019).

## Methods

### Participants

Participants were 67 children; 37 children diagnosed with ASD and 30 TYP children, aged 3–12 years (ASD: *M =* 8.06, *SD* = 0.40; TYP: *M* = 7.41, *SD* = 0.48). Similar to previous studies (DiCriscio et al., 2016; Harrison & Slane, 2020) a broad age range was selected in consideration of the characteristic persistent influence of CIs and reduced attention allocation to social stimuli throughout development in ASD (Frazier et al., 2017; Manyakov et al., 2018). There was no significant difference in age between groups (*t* = -1.06, *p* = .295), however a trend in the distribution of male and female participants was observed, with 66.7% and 89.2% male participants in the TYP and ASD groups, respectively, *χ*^*2*^ (1, 67) = 3.813, *p* = .051, *phi* = 0.024. Participant characteristics are presented in Table 1.

**Table 1:** Participant Characteristics

Characteristic	ASD group (n = 37)			TYP group (n = 30)		
Gender						
Male	33			20		
Female	4			10		
	**Mean**	**SD**	**Range**	**Mean**	**SD**	**Range**
Age in years	8.06	2.42	3–12	7.41	2.63	3–12
Nonverbal IQ^a^	97.24	14.59	68–129	116.04**	12.94	97–151
SRS-2 Mean T-Scores^b^						
Total	77.57	9.36	58–90	46.32**	7.32	36–64
SCI	76.38	9.52	56–90	46.04**	7.21	35–62
RRB^1^	77.89	9.21	48–90	47.79**	9.00	40–80
ADOS-2 CSS						
Total	7.51	1.52	5–10	-	-	-
SA	7.54	1.83	3–10	-	-	-
RRB^2^	7.22	1.89	1–10	-	-	-

ASD, autism spectrum disorder; TYP, typically developing; SRS-2, social responsiveness scale – second edition; SCI, social communication and interaction; RRB^1^, restricted interests and repetitive behaviours; ADOS-2, autism diagnostic observation schedule – second edition; CSS, calibrated severity score; SA, social affect; RRB^2^, repetitive and restricted behaviours.

^a^ Nonverbal IQ scores from the Leiter-3 for participants with ASD (n = 33) and TYP participants (n = 23). ^b^ SRS-2 Mean T-Scores for participants with ASD (n = 37) and TYP participants (n = 28).

** Indicates a significant group difference at *p* < .001.

Participants were recruited through the Clinic for Autism and Neurodevelopmental Research (CAN Research) located at the Brain and Mind Centre, within The University of Sydney. Study eligibility of participants with an ASD diagnosis was confirmed using the Autism Diagnostic Observation Schedule - Second Edition (ADOS-2; Lord C., 2012), administered by research-reliable assessors. Individuals with severe renal, hepatic, cardiovascular or respiratory illness were excluded from the study. Recruitment of TYP participants occurred through locally distributed flyers and by word-of-mouth. Exclusion criteria for TYP participants included neurodevelopmental or mental health diagnoses (e.g., anxiety, depression, ASD, sensory processing disorder) and severe physical illnesses (e.g., severe cardiac, hepatic, renal, respiratory illness).

Once enrolled, all participants were administered the Leiter-3 (Roid et al., 2013), a nonverbal cognitive assessment, and caregivers completed the Social Responsiveness Scale - Second Edition (SRS-2; Constantino & Gruber, 2012) as a global measure of social functioning. The research project was approved by the Human Research Ethic Committee (HREC) of The University of Sydney (references 2013/502, 2013/341), and informed consent was obtained from caregivers prior to study enrolment.(Table 1 top)

### Measures

#### Autism Diagnostic Observation schedule – Second Edition (ADOS-2; Lord C., 2012)

The ADOS-2 is a semi-structured, play-based observational measure of common autism symptoms, which fall under the broad domains of Social Affect (SA; including communication, social interaction, and play-based behaviours) and Repetitive and Restricted Behaviours (RRB; including unusual sensory interests, aggressive and stereotyped behaviours) (Lord et al., 2012). Modules 1 (*n* = 13), 2 (*n* = 15) or 3 (*n* = 9) were administered based on participant age and expressive language level. As a standardised measure of core symptom severity, total and domain calibrated severity scores (CSS), ranging from 1 to 10, were calculated, with higher scores indicating greater symptom severity (Gotham et al., 2009).

#### Leiter International Performance Scale - Third Edition (Leiter-3; Roid et al., 2013)

Designed for assessment of individuals between 3 and 75 years of age, the Leiter-3 is a nonverbal intellectual assessment commonly administered to ASD populations (Roid & Koch, 2017). It comprises 10 subtests which measure cognitive ability across three dimensions, including general IQ, nonverbal memory and processing speed (Roid & Koch, 2017).

#### Social responsiveness scale – Second Edition (SRS-2; Constantino & Gruber, 2012)

The SRS-2 is a 65-item informant-completed rating scale of socially relevant behaviours in ASD that includes both preschool (2:6 to 4:6 years) and school-age (4:0–18:0 years) forms that can be rated by parents and teachers. Items are summed to calculate scores in the domains of Restricted Interests and Repetitive Behaviour (RRB) and Social Communication and Interaction (SCI), which combine to an overall Total Score (Constantino & Gruber, 2012). Results are reported as T-scores, with scores above 75 indicating severe social deficits, scores between of 66 to 75 considered moderate, scores of 60 to 65 considered mild, and scores below 60 indicate no socially challenged behaviour related to ASD (Bruni, 2014).

### Eye-Tracking Task

**Fig. 1:**
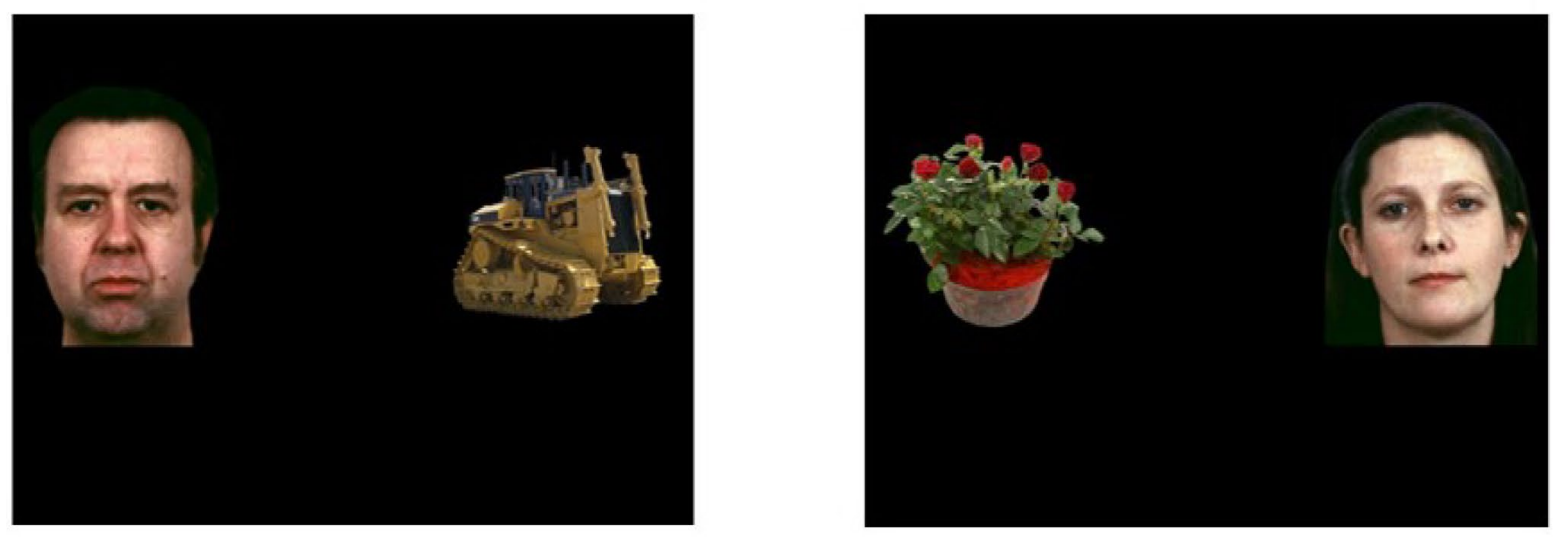
**Example images from the visual preference task developed by Sasson and Touchstone (2014).** (A) Image of a face paired with a HAI; (B) Face paired with a LAI. HAI, high autism interest; LAI, low autism interest

The current study employed the visual preference task developed by Sasson & Touchstone (2014; Fig. 1). The HAI and LAI objects used in this task were previously validated to be of CI and non-CI interest, respectively, across childhood (South et al., 2005). Participants were presented with one block of 20 randomly presented slides of a social image (a face) paired with a HAI (i.e., CI) or LAI object. Neutral, happy, sad, angry, and fearful emotional expressions were each presented four times. Social and object images did not repeat, and their location was counterbalanced between the right and left sides of the screen. Participants sat on a booster chair fitted on a regular office chair, or a regular height-adjustable office chair, and were positioned approximately 65 cm away from a 23-inch computer monitor with a pixel resolution of 1920 × 1080. The monitor was integrated with the Tobii TX300 eye tracker (Tobii Technology, Stockholm, Sweden), which was used to collect eye tracking data with a sampling rate of 300 Hz and spatial accuracy of 0.4 degrees.

Participants completed a 9-point calibration procedure. Once calibration was successfully completed, participants were told they would be looking at some pictures and could look wherever they wanted. Prior to the first paired social and object image slide being presented, an introductory slide reading “Hi, let’s start” was presented to orient attention to the screen. A slide reading “You’re doing really well” was presented half-way through the task to maintain attention to the screen, and a final slide reading “Well done! You’re finished” marked the end of the task. The presenter would read aloud these written prompts as each of these slides were presented to reinforce and tailor the message for participants who were illiterate. Each slide was presented for 5 s, followed by an interstimulus interval (ISI) of an animated figure presented centrally for 500ms to encourage task engagement. The total duration of the task was approximately 1 min and 20 s.

An I-VT filter was applied to raw data, using a velocity threshold of 30 degrees/second and a minimum fixation duration of 60ms (Tobii Technology AB, 2016). Areas of Interest (AOIs) were drawn around each face and object image. Face and object AOIs approximated 15% and 12% of the screen, respectively. Eye tracking variables were aggregated using Tobii Studio Version 3.4.8 (Tobii Technology AB, 2016). Overall, 20 participants did not have eye tracking data collected. Technical issues prevented task administration for 16 participants and calibration could not be completed with four participants due to noncompliance. The data from an additional two participants were excluded from analyses as the quality of their gaze data fell below the 20% threshold adopted in this study, and used in prior studies (for example, Harrop et al., 2018a).

(Fig. 1 top)

### Data Analysis

Similar to previous studies employing this task (Harrop et al., 2018a; Sasson & Touchstone, 2014; Unruh et al., 2016), the following dependent variables (DVs) were analysed as measures of social and object attention: (1) Prioritisation, the latency to first fixate to the face or object; (2) Preference, the proportion of total fixation duration to the face or object relative to total fixation duration to the AOIs on the screen; and (3) Duration, the total fixation duration to the face or object across all trials.

Initial exploratory analyses indicated a non-normal distribution of data for the prioritisation DV only; log transformations were therefore performed, although significant improvements in normality were not observed. As subsequent non-parametric and parametric analyses yielded similar results, parametric results are reported.

Repeated-measures analyses of variance (RM-ANOVAs) were conducted on each DV with object type (HAI, LAI) as the within-group variable, and diagnosis (ASD, TYP) as the between-group variable. Greenhouse-Geisser corrections were applied as the assumption of sphericity was violated.

Exploratory analyses using Spearman’s rank order correlations were conducted between eye-tracking DVs and the ADOS-2 CSS for Total, SA and RRB domains, and with the SRS-2 Total, SCI and RRB domain T-scores. Data were analysed using IBM SPSS Version 26® (IBM Corp, 2019).

## Results

### Preliminary analysis on effect of sex, age, and non-verbal IQ

Given the proportion of male and female participants across both groups, separate univariate analyses of variance (ANOVAs) with sex as the between-subjects factor were conducted on each DV (prioritisation, preference, duration) within both groups. These analyses confirmed no significant effect of sex on any eye-tracking DV within the ASD group (*p* ≥ .271 for all analyses). Likewise, within the TYP group, there was no significant effect of sex on eye tracking variables (p ≥ .070). Age was not included as a covariate due to non-significant differences in mean ages between groups and analyses of scatterplots indicated that the assumption of linearity between age and eye-tracking DVs was not met. Similarly, non-verbal IQ was not included as a covariate as correlational analyses indicated no significant associations with any eye-tracking DV across ASD or TYP groups. Although eligibility criteria did not exclude individuals with lower IQs, only two participants scored below 70 for non-verbal IQ, precluding analysis of differences in gaze behaviour based on this characteristic. Analyses were conducted with and without these participants and the pattern of results remained the same, hence results are reported inclusive of these participants.

### Group differences in eye-tracking variables

#### Prioritisation

##### Faces

**Fig. 2:**
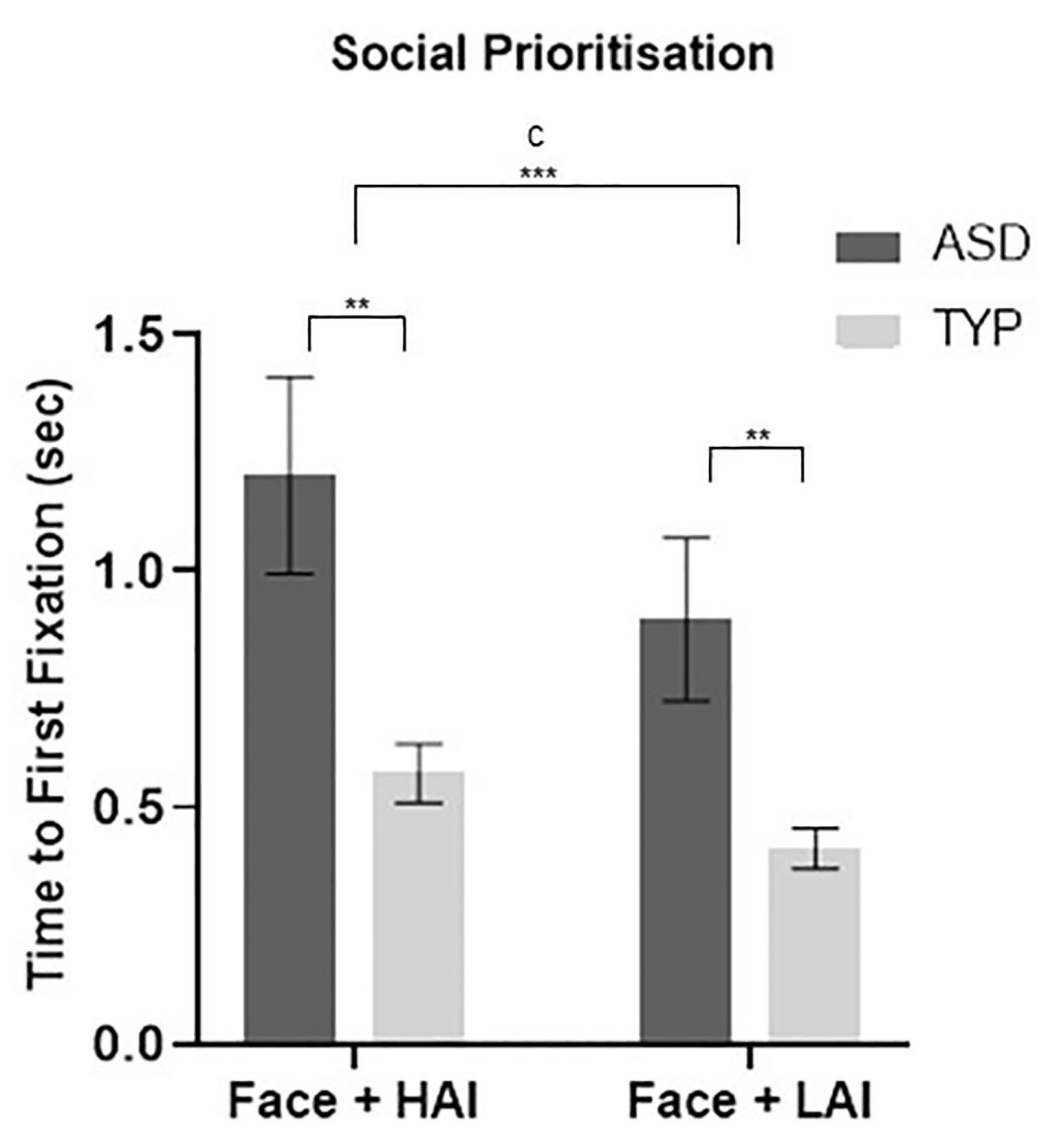
**Mean (+/- standard error) time to first fixation in seconds to faces adjacent to HAI and LAI objects in ASD and TYP groups.** ASD, autism spectrum disorder; TYP, typically developing; HAI, high autism interest; LAI, low autism interest; C, condition effect. *** *p* < 0.001. ** *p* < .05

Results from a 2 × 2 RM-ANOVA indicated no significant object type x diagnosis interaction effect, F(1,64) = 1.799, *p* = .354, *Ƞ*^*2*^_*p*_ = 0.013. There was a significant main effect of object type, F(1, 65) = 8.841, *p* = .004, *Ƞ*^*2*^_*p*_ = 0.120, and a significant main effect of diagnosis, F(1,65) = 7.526, *p* = .008, *Ƞ*^*2*^_*p*_ = 0.104, with the ASD group taking longer to look to the face compared to the TYP group (*M*_*diff*_ = 0.557, *SE* = 0.203, *p* = .008). Group differences for each object type are illustrated in Fig. 2. Post-hoc analyses revealed that both ASD and TYP took longer to prioritise the face when paired with an HAI compared to LAI object (ASD: *M*_*diff*_ = 0.305, *SE* = 0.137, *p* = .033; TYP: *M*_*diff*_ = 0.159, *SE* = 0.037, *p* < .001).

##### Objects

A 2 × 2 RM-ANOVA revealed no significant object type x diagnosis interaction effect F(1,65) = 2.425, *p* = .124, *Ƞ*^*2*^_*p*_ = 0.036. There was a significant main effect of object type F(1,65) = 23.916, *p* < .001, *Ƞ*^*2*^_*p*_ = 0.269. Pairwise comparisons demonstrated that both groups fixated faster to HAI compared to LAI objects (*M*_*diff*_ = 0.314, *SE* = 0.064, *p* < .001). A trend for the main effect of diagnosis, F(1, 65) = 3.938, *p* = .051, *Ƞ*^*2*^_*p*_ = 0.057, indicated the TYP group may have prioritised objects faster than the ASD group (*M*_*diff*_ = 0.393, *SE* = 0.198, *p* = .051). (Fig. 2 top)

### Preference

#### Faces

**Fig. 3:**
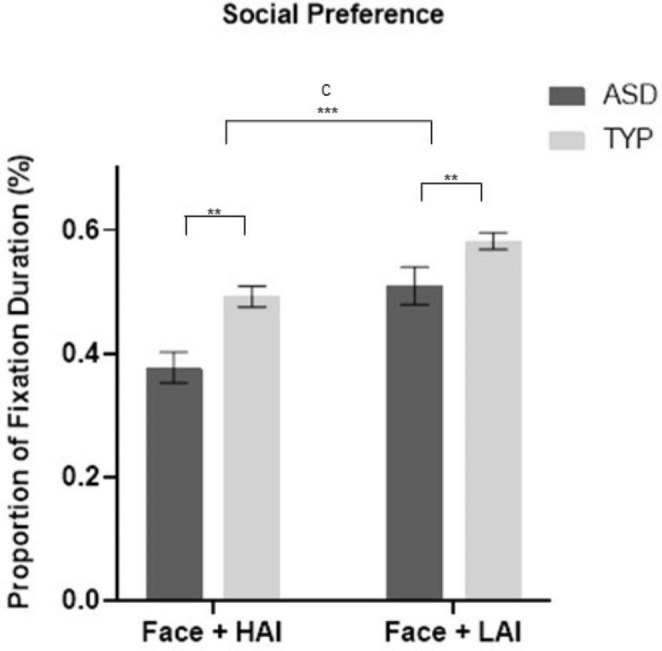
**Mean (+/- standard error) proportion of fixation duration in seconds to faces adjacent to HAI and LAI objects in ASD and TYP groups.** ASD, autism spectrum disorder; TYP, typically developing; HAI, high autism interest; LAI, low autism interest; C, condition effect. *** *p* < .001. ** *p* < .05.

A 2 × 2 RM-ANOVA demonstrated a significant object type x diagnosis interaction effect, F(1,65) = 6.018, *p* = .017, *Ƞ*^*2*^_*p*_ = 0.085 (see Fig. 1 A of Supplementary Information, SI). Significant main effects were found for both object type F(1,65) = 112.397, *p* < .001, *Ƞ*^*2*^_*p*_ = 0.634 and diagnosis F(1,65) = 5.588, *p* = .021, *Ƞ*^*2*^_*p*_ = 0.079. Group differences for both object types are shown in Fig. 3. Overall, the TYP group demonstrated greater preference for faces regardless of object type (*M*_*diff*_ = 0.094, *SE* = 0.031, *p* = .005). Both groups displayed less preference for the face when paired with HAI compared to LAI objects (*M*_*diff*_ = 0.111, *SE* = 0.013, *p* < .001). Follow up analyses indicated that the interaction effect was driven by the TYP group spending significantly more time fixating on faces during HAI trials than the ASD group (*p* = .001), while no significant group difference was evident during LAI trials (*p* = .241).

#### Objects

A 2 × 2 RM-ANOVA resulted in a significant object type x diagnosis interaction effect, F(1,65) = 6.018, *p* = .017, *Ƞ*^*2*^_*p*_ = 0.085 (Fig. 1B of SI), with follow up analyses indicating the ASD group had a significantly greater preference for HAI objects compared to the TYP group, (*p* = .001) while there was no group difference in the preference for LAI objects (*p* = .214). Pairwise comparisons revealed an overall higher preference to view objects in the ASD group (*M*_*diff*_ = 7.047, *SE* = 2.981, *p* = .021), while both groups had a preference to view HAI over LAI objects (*M*_*diff*_ = 13.568, *SE* = 1.280, *p* < .001). (Fig. 3 top)

### Duration

#### Faces

A 2 × 2 RM-ANOVA revealed no significant interaction effects, F(1,65) = 3.144, *p* = .081, *Ƞ*^*2*^_*p*_ = 0.046. Significant main effects were demonstrated for both object type, F(1,65) = 44.013, *p* < .001, *Ƞ*^*2*^_*p*_ = 0.404, and diagnosis, F(1,65) = 44.581, *p* < .001, *Ƞ*^*2*^_*p*_ = 0.407. Pairwise comparisons indicated shorter fixation durations to faces in the presence of HAI objects for both groups (*M*_*diff*_ = 2.672, *SE* = 0.405, *p* < .001), and the TYP group demonstrated longer fixation durations to faces compared to the ASD group (*M*_*diff*_ = 8.944, *SE* = 1.375, *p* < .001).

#### Objects

**Fig. 4:**
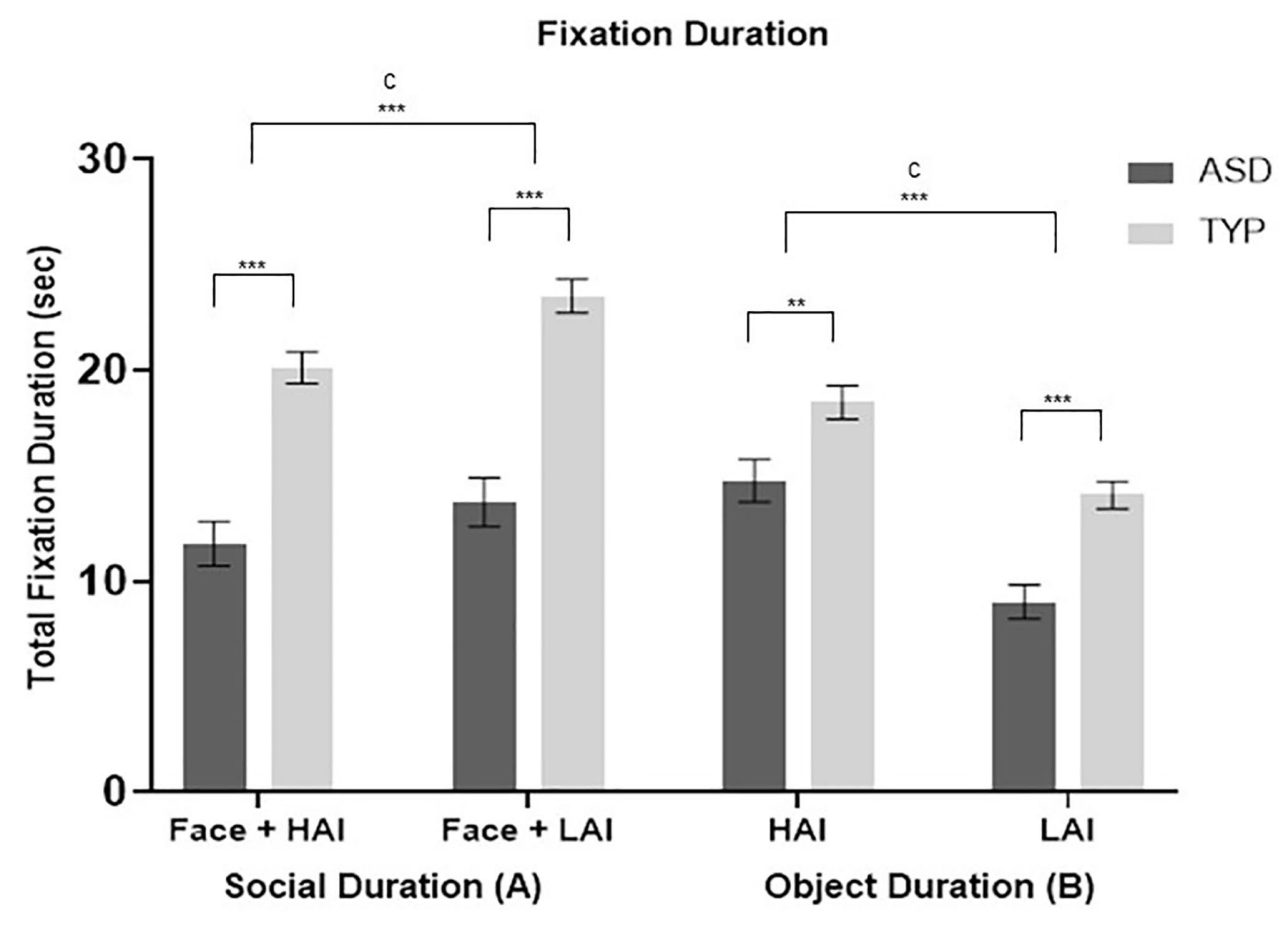
**Mean (+/- standard error) total duration of fixations to face and object stimuli in ASD and TYP groups.** (**A)** Total fixation duration to face adjacent to HAI and LAI objects. **(B)** Total fixation duration to HAI and LAI objects. ASD, autism spectrum disorder; TYP, typically developing; HAI, high autism interest; LAI, low autism interest; C, condition effect. *** *p* < .001. ** *p* < .05.

There was no significant object type x diagnosis interaction for the duration of fixations to objects, F(1,64) = 2.404, *p* = .126, *Ƞ*^*2*^_*p*_ = 0.036. Significant main effects for object type, F(1,65) = 44.013, *p* < .001, *Ƞ*^*2*^_*p*_ = 0.404, and diagnosis, F(1,65) = 44.581, *p* < .001, *Ƞ*^*2*^_*p*_ = 0.407 were found. Pairwise comparisons revealed greater fixation durations to HAI compared to LAI objects for both groups (*M*_*diff*_ = 2.676, *SE* = 0.403, *p* < .001), and the TYP group displayed greater fixation durations to both object types compared to the ASD group (*M*_*diff*_ = 9.064, *SE* = 1.358, *p* < .001). Figure 4 illustrates group differences in fixation duration to both faces and objects.

The means and standard deviations of eye-tracking DVs are detailed in Table 1 of Supplementary Information (SI).

(Fig. 4 top)

### Correlations between eye-tracking variables and clinical measures

Within the ASD group, there were significant medium sized negative correlations between ADOS-2 CSS RRB scores and fixation duration to faces across object types (*ρ* = − 0.339, *p* = .040), and fixation duration to faces adjacent to LAI objects (*ρ* = − 0.401, *p* = .014), indicating that greater attention to faces overall and in the presence of LAI objects, was associated with a lower severity of restricted and repetitive behaviours. A similar correlation was not found for faces adjacent to HAI objects. One additional statistically significant correlation between ADOS-2 Total CSS and fixation duration to HAI objects (*ρ =* − 0.335, *p* = .043) was found, suggesting greater symptom severity was associated with less attention to HAI objects. 

Baseline significant differences in Total, SCI and RRB domain scores of the SRS-2 demonstrated greater impairment in the ASD compared to the TYP group across all three domains. For the ASD group, significant medium sized negative correlations between fixation duration to LAI objects and RRB (*ρ* = − 0.343, *p* = .038), SCI (*ρ* = − 0.353, *p* = .032) and Total scores (*ρ* = − 0.360, *p* = .029) demonstrated that greater attention to LAI objects was associated with higher functioning overall and in behaviours related to social communication and interaction skills, and repetitive behaviours and restricted interests. No significant correlations between attention measures and SRS-2 scores were found for HAI objects or the TYP group. See SI for correlational analyses between eye-tracking DVs and ADOS-2 and SRS-2 scores for the ASD group.

## Discussion

Using an established visual preference eye-tracking task, the purpose of this study was to investigate the contextual influence of circumscribed interests (CIs) on patterns of social and object attention in children with ASD and their TYP peers, and the relationship between these patterns and social functioning more broadly. In support of our first hypothesis, results demonstrated reduced attention to social stimuli in children with ASD compared to TYP children, regardless of the presence of HAI objects (representing CIs). Reduced attention in the ASD group also extended to non-social stimuli, a surprising finding of the study. Analysis of attention patterns indicated that both ASD and TYP groups appeared to be influenced by CIs. That is, both groups exhibited reduced social attention and increased non-social attention in the presence of CIs, in line with our second hypothesis. Among participants with ASD, correlational analyses indicated that greater attention to faces overall and in the presence of LAI objects was related to less severe restricted and repetitive behaviours as measured by the ADOS-2, while greater fixation duration to LAI objects was significantly associated with greater social functioning, as measured by the SRS-2.

Reduced social attention was evident in children with ASD across all three attention measures. Overall, children with ASD were significantly slower in prioritising the face; similarly, their preference to attend and maintain attention to faces was significantly reduced compared to TYP children across object types. Although the contextual influence of CIs did influence the preference to attend to faces in the current study, this finding did not extend to the social prioritisation and duration of attention variables. These results contrast with Sasson & Touchstone (2014) where reduced social attention in pre-schoolers with ASD was only evident when paired with objects related to CIs (i.e., HAIs). The specific influence of CIs on social prioritisation, preference and duration variables led to their conclusion that social attention is influenced by the nature of stimuli competing for attention in this population, rather than a global social saliency deficit as postulated by the social motivation hypothesis (Sasson & Touchstone, 2014). However, the broader reductions in social attention patterns in children with ASD demonstrated in this study are supportive of more recent studies (Harrison & Slane, 2020; Mo et al., 2019; Unruh et al., 2016), and meta-analytic findings of atypical gaze patterns towards social stimuli (Chita-Tegmark, 2016; Frazier et al., 2017), supporting overall reductions in social attention irrespective of the salience of competing stimuli.

Surprisingly, object-directed attention mirrored social attention patterns across both groups of participants. The overall duration of attention to both object types was reduced in children with ASD. Although decreased attention to LAI objects could be reasonably expected (Anderson et al., 2006; Sasson et al., 2011), the finding of overall decreased attention to HAI objects is interesting considering the breadth of literature supporting the increased salience of CIs in this population (Harrop et al., 2018a; Manyakov et al., 2018; Sasson et al., 2011; Sasson & Touchstone, 2014; Traynor et al., 2019; Unruh et al., 2016). A similar trend was observed for the prioritisation of objects, with the TYP group taking a significantly shorter amount of time to fixate to either object type, a result consistent with the other main findings of this study. Reduced object-directed attention in ASD has been reported cross-modally (Keehn et al., 2016, 2017; Parsons et al., 2017). In eye-tracking for example, Parsons et al., (2017) investigated the distribution of attention to object and social stimuli in infants at high and low familial risk of ASD and found that high risk infants later diagnosed with ASD engaged less with objects and this was associated with poorer future vocabulary skills. Supporting this, neurophysiological and neurological evidence of under-reactivity or hypoactivation of attentional networks in response to both social and object stimuli has been replicated across studies, suggesting that differences in attention across social and non-social domains may be implicated in ASD (Clements et al., 2018; Dichter et al., 2010; Keehn et al., 2016, 2017; Richey et al., 2014).

An increased influence of CIs in the attentional patterns of children with ASD was evident in the reduced preference to attend to faces and increased preference to attend to HAI objects in that condition. This finding, which extends the results of Sasson & Touchstone (2014) in toddlers to a broader age cohort across childhood in ASD, suggests that CIs core to ASD do elicit a relative preference to attend to HAI stimuli. However, in this study the influence of CIs was shared across groups with both ASD and TYP children spending less time looking at faces and more time looking at HAI objects. Similarities in the influence of CIs on attention patterns across ASD, TYP and Broad Autism Phenotype (BAP) groups have also been reported in other studies (Goldberg et al., 2017; Morrison et al., 2018; Sasson et al., 2008; Silver et al., 2020). A recent study by Silver et al., (2020), investigated whether children and adults with ASD demonstrated an advantage in the visual processing of CIs and found no differences between ASD and TYP groups in the early visual perception of CIs, but surmised these interests may interfere with later processing streams involved in cognitive control and arousal. These findings also raise questions regarding the contexts and cognitive mechanisms subserving the influence of CIs on atypical attention processes specifically, and socio-cognitive processes and social functioning more generally, in ASD.

The shared influence of CIs across groups and attention variables, and overall reduced attention patterns in the ASD group may also be suggestive of a conceptual and neurocognitive divergence in gaze behaviour to social and CI-related stimuli. Recent studies in both children and adults with ASD and their neurotypical peers have demonstrated that an increased preference for HAI objects was not due to an avoidance of social stimuli (Gale et al., 2019; Manyakov et al., 2018), contributing to the possibility that the increased salience of CIs is conceptually and operationally distinct from the salience of social stimuli such as faces (Bottini, 2018).Although not the focus of this study, abnormalities in the early development and regulation of attentional control and disengagement behaviours have led some researchers to hypothesise a different conceptual framework for understanding shared arousal mechanisms concurrent with atypical attention disengagement and attention shifting mechanisms between social and non-social stimuli in ASD (Keehn et al., 2013). While altered visual processing mechanisms may lead to an overall reduction in attention in ASD (Bellocchi et al., 2017), as seen in this study, the increased salience of CIs under some conditions may partly explain the heterogeneity reported across different studies, providing further impetus for employing multimodal methods investigating underlying neurocognitive process in CI-related visual attention patterns across neurodevelopmental and typically developing cohorts.

In the current study, increased attention allocation to faces overall, and adjacent to LAI objects, was associated with reduced symptom severity in restricted and repetitive behaviours. Furthermore, greater attention to LAI objects directly was associated with higher social skills and milder restricted and repetitive behaviours in children with ASD. While these results appears to be in contrast with previous research highlighting a positive association between social impairment and object-directed attention (Klin et al., 2002; Pierce et al., 2016; Rice et al., 2012), they could potentially reflect that greater task engagement is associated with better social functioning. A similar conclusion was drawn in a social skills intervention study with findings suggesting that higher social functioning in individuals with ASD was associated with increased attention to faces and background objects (Greene et al., 2020). Neurologically, the under-reactivity of biomarkers of arousal and attention has also been associated with atypical attention to behaviourally relevant social and non-social stimuli and ASD symptomatology (Bottini, 2018; Keehn et al., 2016, 2017), providing additional support for the suggestion that both social and non-social visual attention processes may play a complementary role in the social communication and functioning challenges commonly found in individuals with ASD. Due to the small sample size and exploratory nature of the correlational analyses in this study, results should be interpreted with caution and require replication. However, in the future, a larger study investigating task orientation and engagement as a predictor of social functioning and repetitive and restricted behaviours may reinforce some the clinical associations found in this study and disentangle some of the existing heterogeneity in this research area.

There are several other limitations in the present study that warrant consideration. Due to a relatively small sample size, within- and between-group sex differences in social and object attention patterns could not be investigated. As most of the participants in the ASD group were male, and previous research has evidenced similarities in social attention patterns in the presence of CIs in female participants with and without ASD (Harrop et al., 2018a, b), the results of this study may be more applicable to males. Second, the visual preference task administered included object AOIs which shared a relatively smaller proportion of the screen compared to face AOIs. Hence, it is possible that perceptual differences biased differences in attention patterns to social and non-social stimuli, cautioning interpretation of any main effects of object type. As the aim of this study was to investigate the influence of CIs on social and non-social attention patterns, the task was not suited to determine overall reductions in attention. Future studies could incorporate task paradigms designed to probe overall changes or reductions in attention across groups. Additionally, the type of CIs used may have influenced the attention patterns reported in this study. Although the CIs used in this task have been validated across childhood in ASD (South et al., 2005), gender differences in CIs (Harrop et al., 2019; Nowell et al., 2019), and the use of personalised over non-personalised CIs (Harrison & Slane, 2020; Traynor, 2019), have previously been shown to influence social attention patterns in ASD cohorts. The growing number of studies in this area of research suggests a systemic review on the influence of CIs on social attention patterns across childhood in ASD is warranted.

The results of the current study contribute to a growing body of evidence of atypical attention patterns to both social and object stimuli, supportive of the hypothesis that attention atypicalities demonstrated in ASD extend beyond the social domains suggested by the social motivation hypothesis (Bottini, 2018; Chevallier et al., 2012; Clements et al., 2018; Keehn et al., 2016). The results may also suggest that objects common to CIs in children with ASD may play a more general role in influencing their attention patterns and those of their typically developing peers. Indeed, attention to behaviourally or task-relevant stimuli including both social and non-social content may be more instrumental in the day-to-day social and adaptive challenges experienced by children with ASD than is currently understood. Specific to CIs, future cross-modal research, implementing static, as well as dynamic and interactive task paradigms (e.g., Chevallier et al., 2015) may also facilitate a deeper understanding of the operational influence of CIs on early and later stage socio-cognitive processes and how this may change over the course of childhood. Developing a deeper and more robust model of contextual variations to attention patterns throughout development would contribute to our overall understanding of this important behavioural phenotype, and to future research investigating the implications of atypical gaze behaviour in the diagnosis and treatment response of children with ASD.
